# AcrHub: an integrative hub for investigating, predicting and mapping anti-CRISPR proteins

**DOI:** 10.1093/nar/gkaa951

**Published:** 2020-11-02

**Authors:** Jiawei Wang, Wei Dai, Jiahui Li, Qi Li, Ruopeng Xie, Yanju Zhang, Christopher Stubenrauch, Trevor Lithgow

**Affiliations:** Infection and Immunity Program, Biomedicine Discovery Institute and Department of Microbiology, Monash University, VIC 3800, Australia; Infection and Immunity Program, Biomedicine Discovery Institute and Department of Microbiology, Monash University, VIC 3800, Australia; School of Computer Science and Information Security, Guilin University of Electronic Technology, Guilin 541004, China; School of Computer Science and Information Security, Guilin University of Electronic Technology, Guilin 541004, China; School of Computer Science and Information Security, Guilin University of Electronic Technology, Guilin 541004, China; School of Computer Science and Information Security, Guilin University of Electronic Technology, Guilin 541004, China; School of Computer Science and Information Security, Guilin University of Electronic Technology, Guilin 541004, China; Infection and Immunity Program, Biomedicine Discovery Institute and Department of Microbiology, Monash University, VIC 3800, Australia; Infection and Immunity Program, Biomedicine Discovery Institute and Department of Microbiology, Monash University, VIC 3800, Australia

## Abstract

Anti-CRISPR (Acr) proteins naturally inhibit CRISPR-Cas adaptive immune systems across bacterial and archaeal domains of life. This emerging field has caused a paradigm shift in the way we think about the CRISPR-Cas system, and promises a number of useful applications from gene editing to phage therapy. As the number of verified and predicted Acrs rapidly expands, few online resources have been developed to deal with this wealth of information. To overcome this shortcoming, we developed AcrHub, an integrative database to provide an all-in-one solution for investigating, predicting and mapping Acr proteins. AcrHub catalogs 339 non-redundant experimentally validated Acrs and over 70 000 predicted Acrs extracted from genome sequence data from a diverse range of prokaryotic organisms and their viruses. It integrates state-of-the-art predictors to predict potential Acrs, and incorporates three analytical modules: similarity analysis, phylogenetic analysis and homology network analysis, to analyze their relationships with known Acrs. By interconnecting all modules as a platform, AcrHub presents enriched and in-depth analysis of known and potential Acrs and therefore provides new and exciting insights into the future of Acr discovery and validation. AcrHub is freely available at http://pacrispr.erc.monash.edu/AcrHub/.

## Graphical Abstract

**Graphical Abstract:**
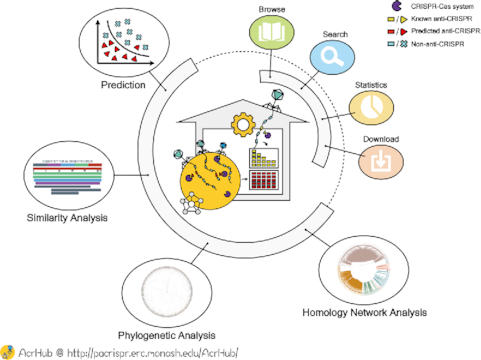
AcrHub: an integrative hub for investigating, predicting and mapping anti-CRISPR proteins.

## INTRODUCTION

Bacteria and archaea have evolved a wide variety of CRISPR-Cas systems to protect themselves from harmful mobile genetic elements (MGEs), such as phages and plasmids (1–4). In response, MGEs evolved a series of potent inhibitors, known as anti-CRISPRs (Acrs), to counteract host CRISPR-Cas defence systems (5–11). Except for their universally short sequences, Acrs have little in common with each other, including very low sequence and structural similarity. At least 50 distinct Acr families have been identified across both bacterial and archaeal domains of life where they each use different molecular mechanisms to inhibit CRISPR-Cas systems (8–10,12–13). Outside the confined environment of a microbial cell, Acrs have inspired a number of downstream applications, from gene editing technologies and protein engineering to phage therapy (10,14–17), applications that are only limited by the relatively small number of known Acr systems compared to the thousands hidden in sequenced genomes.

To overcome this shortfall, several resources were developed either to help categorize Acrs or predict new ones. The three main resources involved in categorizing Acrs are Anti-CRISPRdb (18), which is a compendium of experimentally verified Acrs, the Bondy-Denomy *et al.* online spreadsheet (19) that consolidates Acr records under a uniform naming convention, and AcrCatalog (20), which is a list of predicted Acrs generated using a random forest based model. Alternatively, to identify putative Acrs from within user-defined sequences, there are three online toolkits available. Firstly, AcRanker (21), which ranks phage proteomes for potential Acrs using an eXtreme Gradient Boosting based model. Secondly, AcrFinder, which integrates homology-based, guilt-by-association-based and self-targeting approaches to identify potential Acrs from prokaryotes and phage genomes (22). Thirdly, PaCRISPR (23), which incorporates evolutionary information within an ensemble model to predict Acr proteins from genomic and metagenomic sequences. However, these toolkits are prediction-oriented and have limited downstream capabilities for further exploring those predicted Acrs.

**Figure 1.**
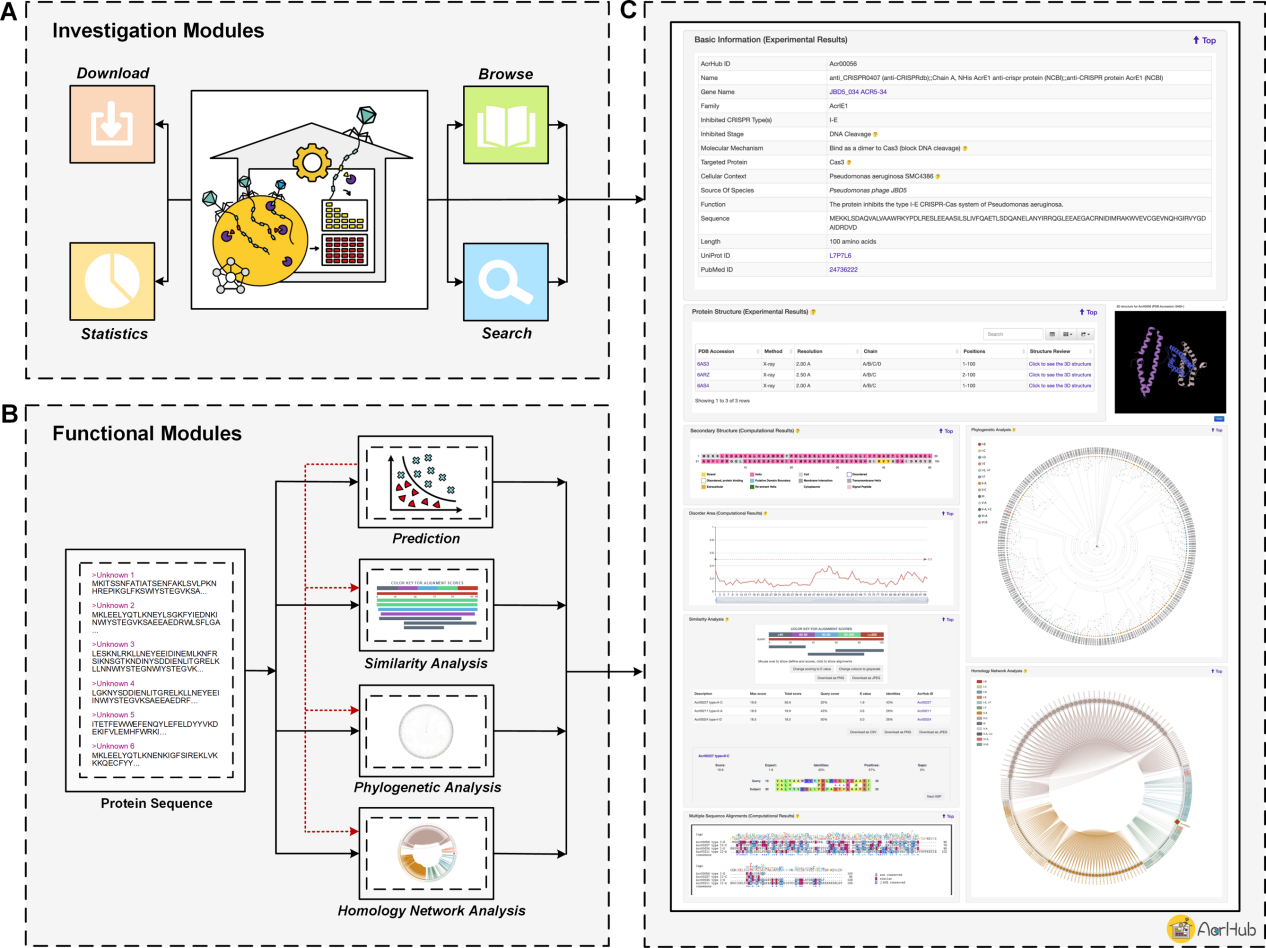
Overview of AcrHub. (**A**) The investigation modules that can be used to examine catalogued Acrs; (**B**) the functional modules and their interactions; (**C**) the ‘Detailed information’ page, using an experimentally validated Acr as example.

Here, we present an integrative platform, AcrHub, to offer an all-in-one, user-friendly solution for Acr protein prediction, characterization and relationship analysis (Figure 1). AcrHub catalogs 339 non-redundant, experimentally validated Acrs and 71 728 putative Acrs, each annotated with their relationships to known Acrs. Additionally, three predictors were integrated into AcrHub for prediction of Acr homologues and novel Acrs: a Hidden Markov Model (HMM) based predictor, AcRanker (21) and PaCRISPR (23). To facilitate relationship analysis between known and potential Acrs, we developed three analytical modules: a BLAST-based similarity analysis, a multiple sequence alignment-based phylogenetic analysis, and a homology-based network analysis. These tools can either work independently or within the AcrHub pipeline to facilitate downstream relationship analysis of a newly predicted Acr and thereby shorten the gap between prediction, functional characterization and eventual experimental validation.

## MATERIALS AND METHODS

Here we present the overall workflow of AcrHub in terms of how we curated and annotated the data, and how we designed and implemented the platform. The architecture of AcrHub incorporates different kinds of modules, including the Acr investigation modules and Acr functional modules (Figure 1).

### Data curation and annotation

We extracted 574 experimentally verified Acrs along with their basic annotations from current literature and available resources, including the Anti-CRISPRdb (18) and unified online Acr spreadsheet (19). After removing redundant sequences, we obtained 339 Acr proteins. Where required, we further manually annotated their attributes and functions from the literature (10,12,14–15,24), including inhibited stages, molecular mechanisms, targeted proteins and the cellular contexts of Acrs (i.e. the species or cell types each Acr has been validated in). Acrs were further annotated with their tertiary structure, derived from the protein data bank (PDB) (25), and their secondary structures using the PSIPRED 4.0 server (26). For each Acr, the disorder area was predicted using the IUPred2A server (27), and visualized by ECharts (https://echarts.apache.org/).

We then retrieved 232 616 non-redundant putative Acrs from AcrCatalog (20). These putative Acrs were extracted from 10 938 430 proteins from the genomes of prokaryotic species and their viruses. Their potential inhibitor types were also annotated according to the CRISPR-Cas types that existed within the original host genomes. Using our PaCRISPR predictor (23) that had been updated on the latest Acr data (described in the following section), we refined our list of 232 616 putative Acrs to 71 728. These putative Acrs were then incorporated into the AcrHub catalogue and were further annotated with their PaCRISPR prediction scores, HMM based prediction results, AcRanker prediction scores and ranks, inhibited CRISPR types, sequences, lengths, accession links to track their original information from the NCBI resource (28) and their predicted disorder areas.

For both experimentally verified and predicted Acrs, we additionally provided multiple pre-calculated relationship analyses to link them to known Acrs. For each entry, the homologous sequences were generated using the BLAST tool (version 2.8.1+) (29) by searching against known Acrs and then were visualized by BlasterJS (30). The R library msa (31) was additionally used to generate and visualize their multiple sequence alignments. For each entry, a phylogenetic tree was generated using FastTree (version 2.1.8) (32) based on the multiple sequence alignments of this entry and known Acrs, which were generated using the MAFFT tool (version v7.271) (33). The open source phylogram_d3 (https://github.com/ConstantinoSchillebeeckx/phylogram_d3) was used to visualize the tree. For each entry, a homology network was generated using the all-against-all BLAST (version blast-2.2.26) (34) on this entry and known Acrs, and then visualized by ECharts. For the linked nodes within the network, their pairwise sequence alignments were generated using EMBOSS Stretcher through its web service (35).

### Functional module implementation

The functional modules of AcrHub are comprised of two types of modules: one predictive module and three analytical modules. The analytical modules were designed to complement the predictive modules and have been implemented in the same manner as detailed within the ‘Data curation and annotation’ section. For the predictive module, we incorporated existing prominent predictors for users to choose from. Designed using different computational techniques, currently existing tools have their own strengths and drawbacks. Implementing a bioinformatic workflow based on well-accepted biological discoveries, AcrFinder (22) accepts nucleotide sequences as input and can provide users with the genomic context of Acrs. However, AcrFinder has limited functionality because it cannot predict Acrs that lack nearby anti-CRISPR-associated genes nor can it predict Acrs in genomes without complete CRISPR-Cas systems. In contrast, machine learning based tools are not restricted in this way, and are usually more accurate in predicting novel Acrs. Among them, AcRanker (21) and the AcrCatalog database method (20) mainly use sequence associated features to predict novel Acrs with similar characteristics to known Acrs. Compared to AcRanker that accepts protein sequences as input, the AcrCatalog method requires a complex matrix input before it will predict Acrs.

An alternative machine learning based predictor is PaCRISPR (23), which was instead trained using position-specific scoring matrix (PSSM) based features. Those high-level features enable PaCRISPR to track the evolutionary history of Acrs, promising a greater capability to predict more distantly related Acrs. However, this potential increase in accuracy comes at the expense of time, due to the requirement to generate PSSMs. We therefore decided to incorporate the more universal and user-friendly prediction tools, AcRanker and PaCRISPR, into the predictive module. AcRanker within AcrHub was implemented using its open-source code and model (21), whereas PaCRISPR within AcrHub was retrained using the same algorithm as the PaCRISPR predictor (23), but with a more up-to-date list of known Acrs. In this way, the evolutionary information derived from the new Acrs was also incorporated into PaCRISPR to expand its detection scope to discover future novel Acrs. Additionally, we developed a lightweight HMM based predictor using HMMER (36) to allow users to rapidly detect homologues of known Acrs from their protein sequences of interest.

### Website architecture

In general, the web interface of AcrHub was displayed by JSP, CSS, jQuery (https://jquery.com/), Bootstrap (https://bootstrapdocs.com/) and their extension packages. The logic actions were controlled by the JAVA (https://www.java.com/) server development suite, including Struts 2 (https://struts.apache.org/) and Hibernate (https://hibernate.org/). All experimentally validated and predicted Acr data was stored in a MySQL database (https://www.mysql.com/).

Specifically, the functional modules introduce an additional Perl CGI (https://metacpan.org/pod/CGI) based server backend to handle those time-consuming operations. Each functional module (implemented as above) was stringed together by a Perl thread within the server end. A Gearman framework (http://gearman.org/) based queueing system was used to decouple the prompt-response-required web interface and the time-consuming server backend for better user experience.

## PROFILING ACRHUB

**Figure 2.**
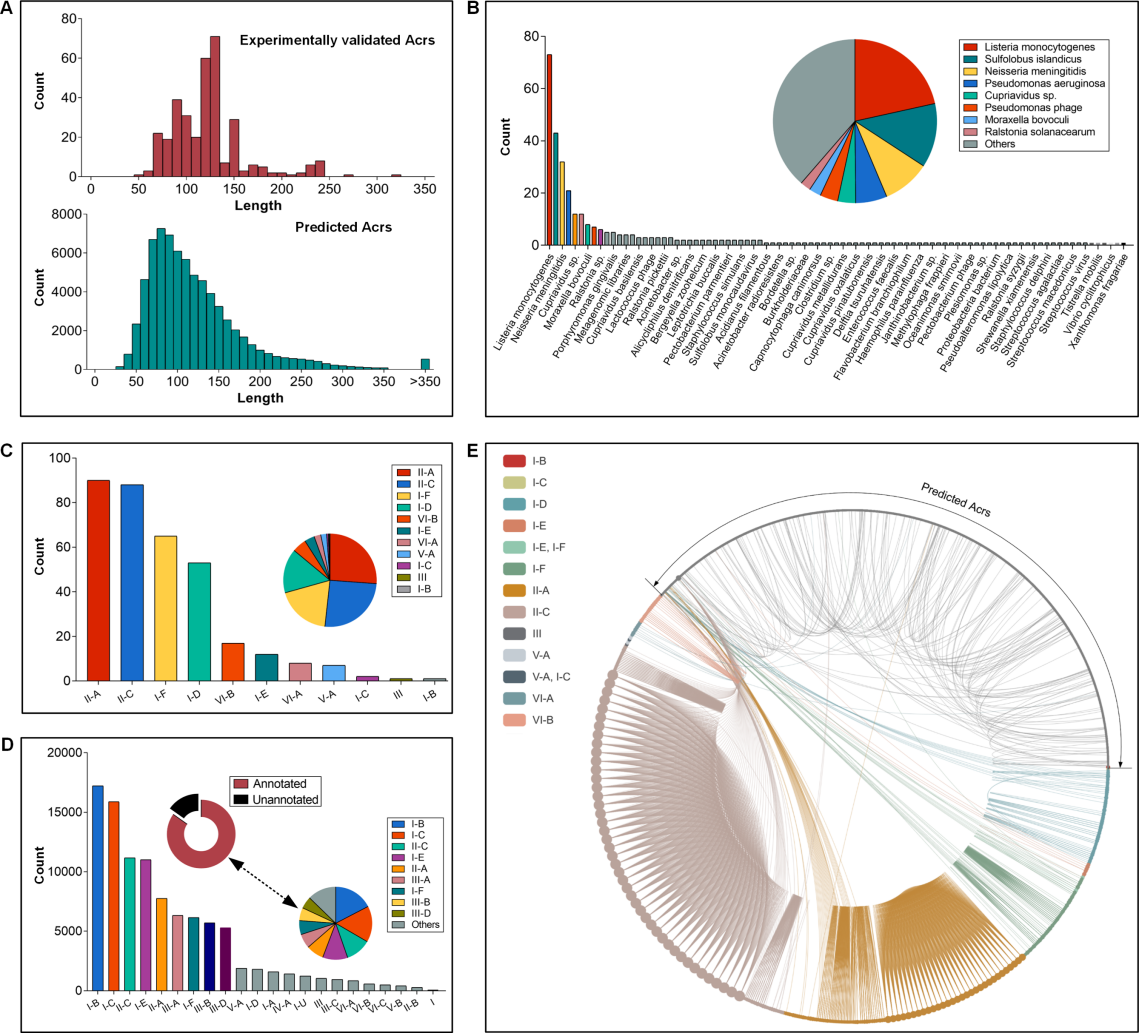
Acr Statistics. (**A**) Distribution of 339 experimentally verified Acrs and 71 728 predicted Acrs by their sequence lengths. (**B**) Distribution of 339 experimentally verified Acrs by their origin. The histogram chart counts the exact numbers, while the pie chart shows the percentage. This applies to panels C and D. (**C**) Distribution of 339 experimentally verified Acrs in terms of their inhibited CRISPR types. The Acrs with two CRISPR types inhibited were counted twice, once for each inhibited CRISPR type. (**D**) Distribution of 71 728 predicted Acrs in terms of their possible inhibited CRISPR types. As there are 10 902 predicted Acrs not yet annotated (denoted by black circle segment), only 60 826 with possible inhibited CRISPR types were counted. The predicted Acrs with more than one possible CRISPR types inhibited are counted for each of their inhibited CRISPR types. (**E**) Homology network of 339 experimentally verified Acrs and 71 728 predicted Acrs. The experimentally verified Acrs are grouped and colored by their inhibited CRISPR types, while the predicted Acrs are indicated by an arc. Each node represents an Acr or a predicted Acr protein. A link between two nodes represents they have a homology relationship. The more links a node has, the larger the node will be. This leads to the unequal distribution across the circle, with the much smaller number of validated Acrs accounting for a larger proportion of the circle. All statistics are reflected in the interactive visualizations in the ‘Statistics’ page of AcrHub.

AcrHub contains a browsable list of the known, experimentally validated Acrs and a vast number of pre-calculated potential Acrs, making it possible to conduct systematic, comparative analyses to enhance current understanding and facilitate future discovery. A majority of experimentally validated Acrs are fewer than 150 amino acids in length (Figure 2A), while the largest is comprised of 322 amino acids: AcrVA2 (AcrHub ID: Acr00311), identified from a prophage and inhibiting the V-A CRISPR-Cas system. Likewise, predicted Acrs are small molecules, where the majority of Acrs were smaller than 150 amino acids (Figure 2A). To date (as of September 2020), 11 inhibited CRISPR types have been confirmed in Acrs (Figure 2C), which has been identified from multiple origins, including prokaryotes, phages and prophages (Figure 2B). Even from our list of predicted Acrs catalogued in AcrHub, there are novel types of CRISPR-Cas systems that are possibly inhibited (Figure 2D). In most cases, homologs exist within the same inhibited CRISPR types due to additional related homolog retrieval applied after a novel Acr was discovered (Figure 2E). Few links exist across different inhibited CRISPR types, highlighting their low sequence similarity. Looking into the predicted Acrs (Figure 2E), we can see some of them have links to known Acrs, which indicates that they might have similar inhibited CRISPR types or functions. On the other hand, most of the predicted Acrs have no links to each other and only very few links within the predicted Acrs (Figure 2E). This promises the future discovery of novel Acrs with distinct functions.

## USING ACRHUB

**Figure 3.**
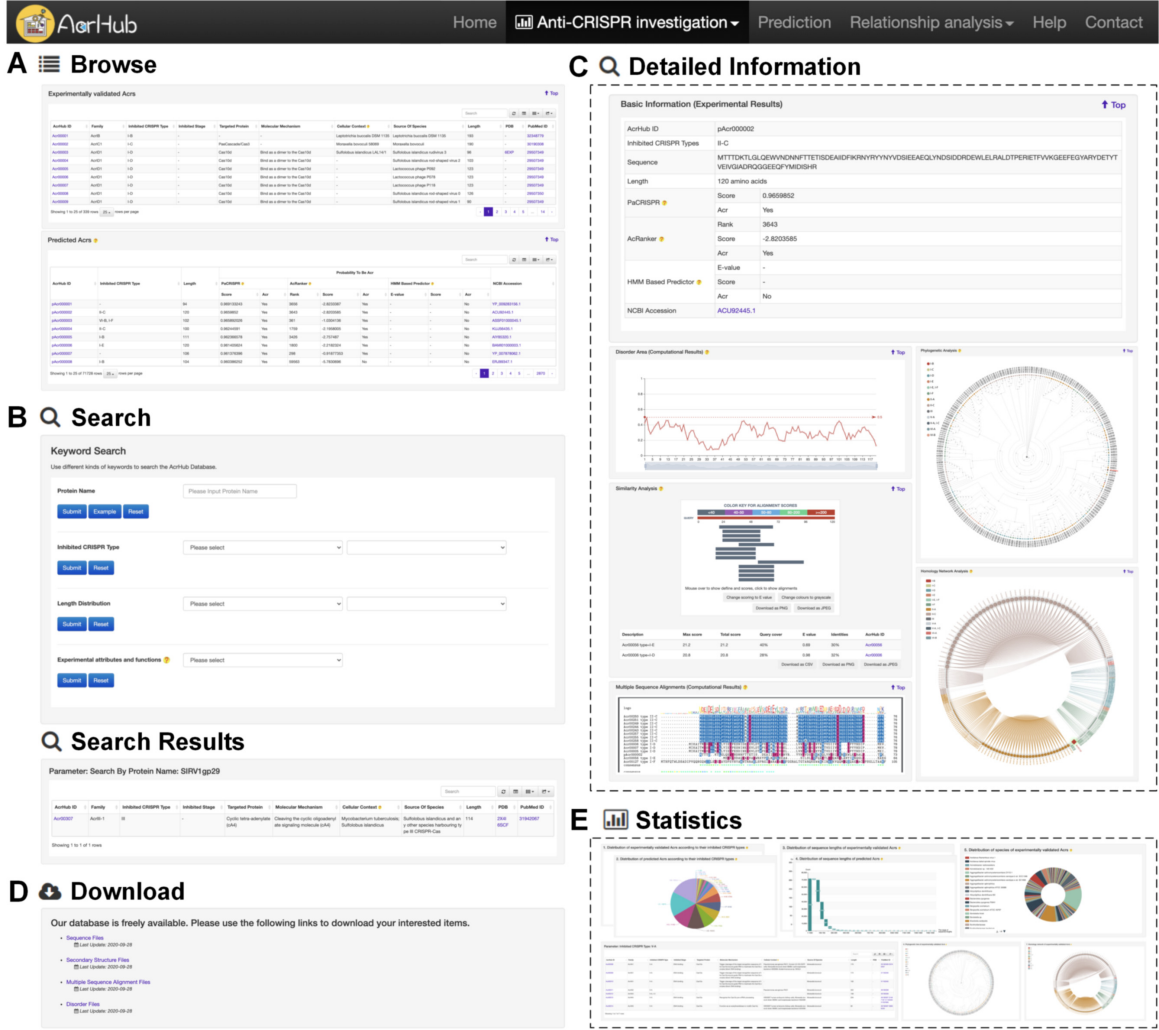
Graphical illustration of the investigation modules in AcrHub: (**A**) the ‘Browse’ page, (**B**) the ‘Search’ and ‘Search results’ pages, (**C**) the ‘Detailed information’ page to describe a predicted Acr protein, (**D**) the ‘Download’ page and (**E**) the ‘Statistics’ page.

**Figure 4.**
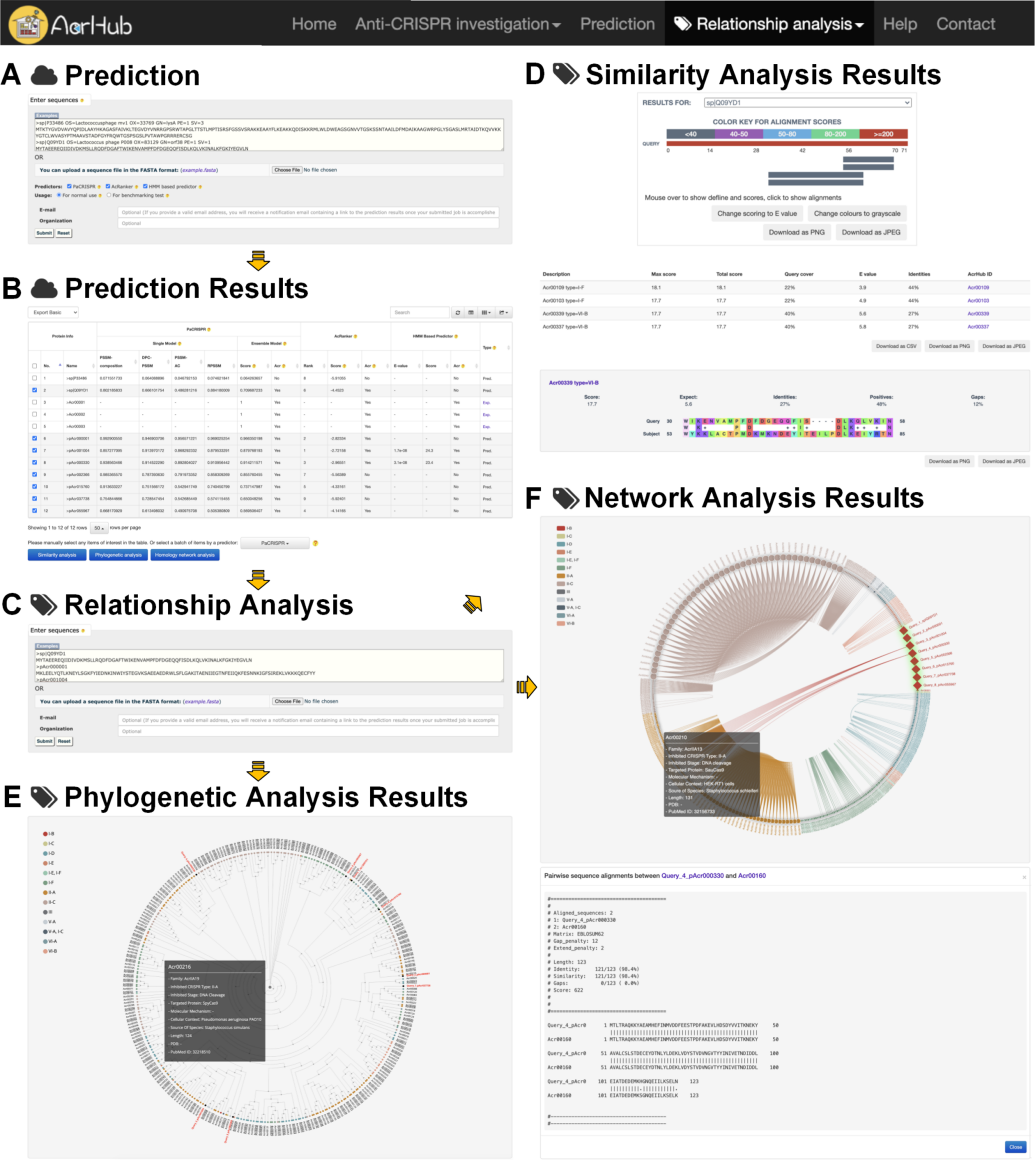
Graphical illustration of the functional modules in AcrHub, from (**A** and **B**) the Prediction input and results pages, (**C**-**F**) to the Relationship analysis input and results pages.

The AcrHub platform includes *Home*, *Anti-CRISPR investigation*, *Prediction*, *Relationshipanalysis*, *Help* and *Contact* modules (Figures 1, 3 and 4). Within *Anti-CRISPR investigation*, AcrHub provides its investigation modules to explore its list of 339 experimentally verified proteins and 71 728 predicted proteins with *Browse*, *Search*, *Statistics* and *Download* functions included (Figures 1 and 3). The *Prediction* module provides users with state-of-the-art predictors to better assist users in predicting new Acrs using their own query sequences. After successful prediction of a new Acr, users can select from three visualization tools to explore their data, or they can access those modules independently under *Relationshipanalysis*: *Similarity analysis*, *Phylogenetic analysis* and *Homology network analysis* (Figures 1 and 4). All of these tools and options are described within the *Help* module, which includes a detailed overview of AcrHub.

### Using investigation modules to explore known and predicted Acrs

The Acr investigation modules allow users to browse, search, download and explore statistics and details of 339 known and 71 728 predicted Acrs stored within AcrHub (Figures 1 and 3), and is found within the ‘Anti-CRISPR investigation’ tab.

*Browsing*. The ‘Browse’ page presents the list of experimentally validated and predicted Acrs. Experimentally validated Acrs are categorized by the CRISPR type that is inhibited, and summarized with their AcrHub IDs, families, inhibited CRISPR types, inhibited stages, targeted proteins, molecular mechanisms, cellular contexts, sources of species, lengths, PDB accessions and PubMed IDs (28). Predicted Acrs are sorted by their PaCRISPR prediction scores, and summarized with their AcrHub IDs, inhibited CRISPR types, lengths, NCBI resource accession numbers and prediction results from PaCRISPR, AcRanker and the HMM based predictor.
*Searching*. The ‘Search’ page allows users to search for known or predicted Acrs using AcrHub ID, UniProt ID, possible protein name (smart partial match supported), inhibited CRISPR type and specified length. For users who have a particular interest in Acrs with experimental attributes and functions, we provide a search function to allow users to specify an option, including protein structure, inhibited stage, molecular mechanism, targeted protein and cellular context. The ‘Search results’ page has an output format similar to that described for the ‘Browse’ page, but filtered according to the search options.
*Detailed information*. The ‘Detailed information’ page opens when a user selects one of the listed Acrs or predicted Acrs. The page provides all of the relevant annotations for that entry. For each of the known Acrs (Figure 1C), experimental annotations (where available) are: its name (as complete as possible), gene name, family, inhibited CRISPR type(s), inhibited stage, molecular mechanism, targeted protein, cellular context, source of species, function, sequence, length, UniProt ID to trace the original sequence information and PubMed ID to trace the original reference. Experimentally confirmed protein structures are provided where available, including PDB accession numbers to track more comprehensive annotations therein and a link to trigger a popup for an interactive three-dimensional structure. A predicted visualization of secondary structure is also presented. For each of the 71 728 predicted Acrs within AcrHub (Figure 3C), annotations include possible inhibited CRISPR types, amino acid sequence and length, and its prediction results, which indicate the possibility of being an Acr. A link to its entry within the NCBI resource is also provided for more comprehensive annotations. For both experimentally verified and predicted Acrs (Figures 1C and 3C), AcrHub presents predicted disorder areas and pre-calculated relationship analyses against known Acrs in AcrHub, including sequence similarity, multiple sequence alignments, phylogenetic relationships and homology networks. For each Acr, this allows users to identify the closest related Acr and provides links to access those known Acrs for more information.
*Statistics*. The ‘Statistics’ page provides interactive visualizations on the statistics of AcrHub catalogued Acrs (validated and putative) (Figure 3E). These include phylogenetic tree and homology network analyses, as well as protein distributions according to their (i) inhibited CRISPR types, (ii) sequence lengths and (iii) species of origin. Users can hover over any node of interest in the phylogenetic tree or homology network to browse the summary information for that Acr. Alternatively, users can click the node of interest to investigate the detailed information for that Acr. Users can also click any link in the homology network to view the pairwise sequence alignment between the two linked Acrs. Similarly, users can click on a bar or part of a pie chart to obtain the corresponding list of Acrs, which is displayed similarly to the ‘Search results’ page.
*Downloading the data*. The ‘Download’ page provides multiple options for users to download the database items, including sequences and annotations of experimentally validated and predicted Acrs (Figure 3D). For experimentally validated Acrs, we additionally provide predicted secondary structures, predicted disorder files and predicted multiple sequence alignments.

### Using functional modules to explore potential Acrs

The functional modules allow users to predict novel Acrs and analyze their relationship to known Acrs listed in AcrHub (Figures 1 and 4). These modules can run independently and allow a maximum of 5 000 sequences per submission.

*Prediction*. AcrHub provides novel Acr predictive functions by incorporating PaCRISPR, AcRanker and an HMM based predictor. Accepting protein sequences as input, PaCRISPR and AcRanker can predict novel Acrs whereas the HMM based predictor can identify very close homologs to known Acrs. Users are provided with a checkbox so they can select one or more of the three predictors to customize different prediction scenarios (Figure 4A). Submitted sequences will first be checked against the experimentally validated Acrs, and those with successful hits will be marked with ‘Exp.’ (i.e. Experimentally verified) and links to their ‘Detailed information’ pages (Figure 4B). The rest of the sequences will be sent to the prediction engine and prediction results of the selected predictor(s) will be retrieved.
*Similarity analysis*. The ‘Similarity analysis’ module of AcrHub aims to find regions of similarity between the query proteins and experimentally validated Acrs (Figure 4C). For each of the query sequences, AcrHub will visualize its known Acr homologs in terms of alignment scores, and list the detailed alignment information, including accurate alignment scores, query cover, *E*-value, identities and AcrHub ID (Figure 4D). Clicking any of the experimentally validated homologs will turn to its pairwise sequence alignment against the query sequence. Clicking any of the AcrHub IDs will redirect users to the ‘Detailed information’ page for that Acr.
*Phylogenetic analysis*. The ‘Phylogenetic analysis’ module aims to recognize the closest phylogenetic relationships of query proteins with experimentally validated Acrs (Figure 4C). As a result, AcrHub presents an interactive phylogenetic tree, with the experimentally validated Acrs categorized by their inhibited CRISPR types and the query proteins highlighted in red (Figure 4E). Users can hover over any protein of interest surrounding the query proteins to browse the summary information for that Acr, or click it to be redirected to its ‘Detailed information’ page.
*Homology network analysis*. The ‘Homology network’ analysis module aims to recognize the closest homologous relationships between query proteins and experimentally validated Acrs (Figure 4C). As a result, AcrHub presents an interactive homology network (Figure 4F): the nodes are categorized by their inhibited CRISPR types, the inquiry proteins are highlighted by red diamonds and links between two nodes indicates their close relationship. Users can hover over any node of interest to browse the summary information for that Acr, click it to be redirected to the ‘Detailed information’ page for that Acr, or click any link between two nodes (if available) to display their pairwise sequence alignment information.

### Analyzing data within the pipeline

The functional modules can also run within a pipeline (Figures 1 and 4). AcrHub provides options for users to redirect prediction results to relationship analysis tools easily, as well as links to ‘Detailed information’ pages for known Acrs homologous to the query proteins within each functional module.

*From prediction to relationship analysis*. Following successful prediction of novel Acr proteins, users have the option to further analyze their relationship to known Acrs. This will provide insight into the possible CRISPR types inhibited or functions of the predicted Acrs. Accordingly, the ‘Prediction results’ page of AcrHub provides an option for users to select their proteins using a specified prediction threshold and redirects them to any of the three relationship analysis modules (Figure 4B and C). This batch operation largely saves time, especially when screening a subset of Acrs from a much larger query.
*From computational results to known Acrs*. The functional modules interact well with the investigation modules (Figure 1). Within the prediction or relationship analysis results (Figure 4B and D-F), links are available that will direct users to the ‘Detailed information’ pages of known Acrs (with homology to the query proteins). This takes full advantage of the well-annotated known Acrs listed in AcrHub.

## DISCUSSION

Deeper understanding of Acrs promises a number of real-world applications and exciting insights into the mechanisms of CRISPR-Cas systems, phage biology, and host–pathogen interactions. We therefore developed AcrHub, an integrative platform that consolidates the most up-to-date list of known and putative Acrs, while providing access to prediction and relationship analysis tools. All modules work independently or within the AcrHub pipeline to better assist biologists with extracting the data they need to help design experiments and formulate interesting hypotheses.

AcrHub will be systematically updated to include newly identified Acrs and the most up-to-date information for existing Acrs. Keeping track of the community efforts in Acr prediction, we will continue to incorporate new Acr predictors and update existing predictors once they become available. Additionally, our PaCRISPR and HMM based tools will be periodically retrained with the rapidly accumulating Acr data. Newly predicted Acrs will also be incorporated into AcrHub by executing PaCRISPR on the ever-expanding volume of prokaryotic and phage genomes. One of the key features of AcrHub is its relationship analysis modules that allow users to visualize their data, so AcrHub will naturally incorporate additional analysis tools as new visualization programs become available or if there is a clear need as defined by users.

## DATA AVAILABILITY

The AcrHub platform is freely available at http://pacrispr.erc.monash.edu/AcrHub/. All data indexed by AcrHub can be downloaded via http://pacrispr.erc.monash.edu/AcrHub/download.jsp. Detailed user instructions can be accessed via the ‘Help’ page at http://pacrispr.erc.monash.edu/AcrHub/help.jsp.

## References

[1] Makarova K.S., Wolf Y.I., Alkhnbashi O.S., Costa F., Shah S.A., Saunders S.J., Barrangou R., Brouns S.J., Charpentier E., Haft D.H. et al. An updated evolutionary classification of CRISPR-Cas systems. Nat. Rev. Microbiol. 2015; 13:722–736. 26411297 10.1038/nrmicro3569 PMC5426118

[2] Sorek R., Lawrence C.M., Wiedenheft B. CRISPR-mediated adaptive immune systems in bacteria and archaea. Annu. Rev. Biochem. 2013; 82:237–266. 23495939 10.1146/annurev-biochem-072911-172315

[3] Barrangou R., Marraffini L.A. CRISPR-Cas systems: prokaryotes upgrade to adaptive immunity. Mol. Cell. 2014; 54:234–244. 24766887 10.1016/j.molcel.2014.03.011 PMC4025954

[4] Amitai G., Sorek R. CRISPR-Cas adaptation: insights into the mechanism of action. Nat. Rev. Microbiol. 2016; 14:67–76. 26751509 10.1038/nrmicro.2015.14

[5] Bondy-Denomy J., Pawluk A., Maxwell K.L., Davidson A.R. Bacteriophage genes that inactivate the CRISPR/Cas bacterial immune system. Nature. 2013; 493:429–432. 23242138 10.1038/nature11723 PMC4931913

[6] Borges A.L., Davidson A.R., Bondy-Denomy J. The discovery, mechanisms, and evolutionary impact of anti-CRISPRs. Annu. Rev. Virol. 2017; 4:37–59. 28749735 10.1146/annurev-virology-101416-041616 PMC6039114

[7] Maxwell K.L. The anti-CRISPR story: a battle for survival. Mol. Cell. 2017; 68:8–14. 28985512 10.1016/j.molcel.2017.09.002

[8] Pawluk A., Davidson A.R., Maxwell K.L. Anti-CRISPR: discovery, mechanism and function. Nat. Rev. Microbiol. 2018; 16:12–17. 29062071 10.1038/nrmicro.2017.120

[9] Stanley S.Y., Maxwell K.L. Phage-encoded anti-CRISPR defenses. Annu. Rev. Genet. 2018; 52:445–464. 30208287 10.1146/annurev-genet-120417-031321

[10] Davidson A.R., Lu W.T., Stanley S.Y., Wang J., Mejdani M., Trost C.N., Hicks B.T., Lee J., Sontheimer E.J. Anti-CRISPRs: protein inhibitors of CRISPR-Cas systems. Annu. Rev. Biochem. 2020; 89:309–332. 32186918 10.1146/annurev-biochem-011420-111224 PMC9718424

[11] Peng X., Mayo-Munoz D., Bhoobalan-Chitty Y., Martinez-Alvarez L. Anti-CRISPR proteins in archaea. Trends Microbiol. 2020; 28:913–921. 32499102 10.1016/j.tim.2020.05.007

[12] Hardouin P., Goulet A. Diversity of molecular mechanisms used by anti-CRISPR proteins: the tip of an iceberg?. Biochem. Soc. Trans. 2020; 48:507–516. 32196554 10.1042/BST20190638

[13] Wiegand T., Karambelkar S., Bondy-Denomy J., Wiedenheft B. Structures and strategies of anti-CRISPR-mediated immune suppression. Annu. Rev. Microbiol. 2020; 74:21–37. 32503371 10.1146/annurev-micro-020518-120107 PMC7712631

[14] Marino N.D., Pinilla-Redondo R., Csorgo B., Bondy-Denomy J. Anti-CRISPR protein applications: natural brakes for CRISPR-Cas technologies. Nat. Methods. 2020; 17:471–479. 32203383 10.1038/s41592-020-0771-6 PMC8510557

[15] Liu Q., Zhang H., Huang X. Anti-CRISPR proteins targeting the CRISPR-Cas system enrich the toolkit for genetic engineering. FEBS J. 2020; 287:626–644. 31730297 10.1111/febs.15139

[16] Basgall E.M., Goetting S.C., Goeckel M.E., Giersch R.M., Roggenkamp E., Schrock M.N., Halloran M., Finnigan G.C. Gene drive inhibition by the anti-CRISPR proteins AcrIIA2 and AcrIIA4 in Saccharomyces cerevisiae. Microbiology. 2018; 164:464–474. 29488867 10.1099/mic.0.000635 PMC5982135

[17] Bubeck F., Hoffmann M.D., Harteveld Z., Aschenbrenner S., Bietz A., Waldhauer M.C., Borner K., Fakhiri J., Schmelas C., Dietz L. et al. Engineered anti-CRISPR proteins for optogenetic control of CRISPR-Cas9. Nat. Methods. 2018; 15:924–927. 30377362 10.1038/s41592-018-0178-9

[18] Dong C., Hao G.F., Hua H.L., Liu S., Labena A.A., Chai G., Huang J., Rao N., Guo F.B. Anti-CRISPRdb: a comprehensive online resource for anti-CRISPR proteins. Nucleic Acids Res. 2018; 46:D393–D398. 29036676 10.1093/nar/gkx835 PMC5753274

[19] Bondy-Denomy J., Davidson A.R., Doudna J.A., Fineran P.C., Maxwell K.L., Moineau S., Peng X., Sontheimer E.J., Wiedenheft B. A unified resource for tracking anti-CRISPR names. CRISPR J. 2018; 1:304–305. 31021273 10.1089/crispr.2018.0043 PMC10625466

[20] Gussow A.B., Park A.E., Borges A.L., Shmakov S.A., Makarova K.S., Wolf Y.I., Bondy-Denomy J., Koonin E.V. Machine-learning approach expands the repertoire of anti-CRISPR protein families. Nat. Commun. 2020; 11:3784. 32728052 10.1038/s41467-020-17652-0 PMC7391736

[21] Eitzinger S., Asif A., Watters K.E., Iavarone A.T., Knott G.J., Doudna J.A., Minhas F. Machine learning predicts new anti-CRISPR proteins. Nucleic Acids Res. 2020; 48:4698–4708. 32286628 10.1093/nar/gkaa219 PMC7229843

[22] Yi H., Huang L., Yang B., Gomez J., Zhang H., Yin Y. AcrFinder: genome mining anti-CRISPR operons in prokaryotes and their viruses. Nucleic Acids Res. 2020; 48:W358–W365. 32402073 10.1093/nar/gkaa351 PMC7319584

[23] Wang J., Dai W., Li J., Xie R., Dunstan R.A., Stubenrauch C., Zhang Y., Lithgow T. PaCRISPR: a server for predicting and visualizing anti-CRISPR proteins. Nucleic Acids Res. 2020; 48:W348–W357. 32459325 10.1093/nar/gkaa432 PMC7319593

[24] Trasanidou D., Geros A.S., Mohanraju P., Nieuwenweg A.C., Nobrega F.L., Staals R.H.J. Keeping CRISPR in check: diverse mechanisms of phage-encoded anti-CRISPRS. FEMS Microbiol. Lett. 2019; 366:fnz098. 31077304 10.1093/femsle/fnz098 PMC6538845

[25] Burley S.K., Berman H.M., Bhikadiya C., Bi C., Chen L., Di Costanzo L., Christie C., Dalenberg K., Duarte J.M., Dutta S. et al. RCSB Protein Data Bank: biological macromolecular structures enabling research and education in fundamental biology, biomedicine, biotechnology and energy. Nucleic Acids Res. 2019; 47:D464–D474. 30357411 10.1093/nar/gky1004 PMC6324064

[26] Buchan D.W.A., Jones D.T. The PSIPRED protein analysis Workbench: 20 years on. Nucleic Acids Res. 2019; 47:W402–W407. 31251384 10.1093/nar/gkz297 PMC6602445

[27] Meszaros B., Erdos G., Dosztanyi Z. IUPred2A: context-dependent prediction of protein disorder as a function of redox state and protein binding. Nucleic Acids Res. 2018; 46:W329–W337. 29860432 10.1093/nar/gky384 PMC6030935

[28] Sayers E.W., Beck J., Brister J.R., Bolton E.E., Canese K., Comeau D.C., Funk K., Ketter A., Kim S., Kimchi A. et al. Database resources of the National Center for Biotechnology Information. Nucleic Acids Res. 2020; 48:D9–D16. 31602479 10.1093/nar/gkz899 PMC6943063

[29] Camacho C., Coulouris G., Avagyan V., Ma N., Papadopoulos J., Bealer K., Madden T.L. BLAST+: architecture and applications. BMC Bioinformatics. 2009; 10:421. 20003500 10.1186/1471-2105-10-421 PMC2803857

[30] Blanco-Miguez A., Fdez-Riverola F., Sanchez B., Lourenco A. BlasterJS: a novel interactive JavaScript visualisation component for BLAST alignment results. PLoS One. 2018; 13:e0205286. 30300406 10.1371/journal.pone.0205286 PMC6177166

[31] Bodenhofer U., Bonatesta E., Horejs-Kainrath C., Hochreiter S. msa: an R package for multiple sequence alignment. Bioinformatics. 2015; 31:3997–3999. 26315911 10.1093/bioinformatics/btv494

[32] Price M.N., Dehal P.S., Arkin A.P. FastTree 2–approximately maximum-likelihood trees for large alignments. PLoS One. 2010; 5:e9490. 20224823 10.1371/journal.pone.0009490 PMC2835736

[33] Katoh K., Misawa K., Kuma K., Miyata T. MAFFT: a novel method for rapid multiple sequence alignment based on fast Fourier transform. Nucleic Acids Res. 2002; 30:3059–3066. 12136088 10.1093/nar/gkf436 PMC135756

[34] Altschul S.F., Madden T.L., Schaffer A.A., Zhang J., Zhang Z., Miller W., Lipman D.J. Gapped BLAST and PSI-BLAST: a new generation of protein database search programs. Nucleic Acids Res. 1997; 25:3389–3402. 9254694 10.1093/nar/25.17.3389 PMC146917

[35] Madeira F., Park Y.M., Lee J., Buso N., Gur T., Madhusoodanan N., Basutkar P., Tivey A.R.N., Potter S.C., Finn R.D. et al. The EMBL-EBI search and sequence analysis tools APIs in 2019. Nucleic Acids Res. 2019; 47:W636–W641. 30976793 10.1093/nar/gkz268 PMC6602479

[36] Potter S.C., Luciani A., Eddy S.R., Park Y., Lopez R., Finn R.D. HMMER web server: 2018 update. Nucleic Acids Res. 2018; 46:W200–W204. 29905871 10.1093/nar/gky448 PMC6030962

